# The BLTT (Bonn Leistungs Tracking Test) in Patients with Temporal Lobe Gliomas—Feasibility of a Novel Neurocognitive Test Battery

**DOI:** 10.3390/neurosci7040091

**Published:** 2026-08-19

**Authors:** Sarah-Marie Gallert, Julia Taube, Anna-Laura Potthoff, Thomas Zeyen, Valeri Borger, Motaz Hamed, Rainer Surges, Hartmut Vatter, Christoph Helmstaedter, Matthias Schneider

**Affiliations:** 1 Clinic and Polyclinic for Neurosurgery, University Hospital Bonn, University of Bonn, 53127 Bonn, Germanymatthias.schneider@ukbonn.de (M.S.); 2 Tumor Translational Research Group, University Hospital Bonn, University of Bonn, 53127 Bonn, Germany; 3 Clinic and Polyclinic for Epileptology, University Hospital Bonn, University of Bonn, 53127 Bonn, Germany; 4 Clinic for Neuro-Oncology, Center for Neurology and Integrated Oncology (CIO), University Hospital Bonn, University of Bonn, 53127 Bonn, Germany

**Keywords:** temporal glioma, high-grade glioma, cognition, neurocognitive assessment, feasibility, Bonn Leistungs Tracking Test (BLTT)

## Abstract

Background: Neurocognitive testing in neuro-oncological patients often relies on time-intensive batteries with limited feasibility in this patient cohort. We evaluated the feasibility of the Bonn Leistungs Tracking Test (BLTT), a time-efficient screening tool assessing episodic memory, semantic memory, and executive functions in patients with high-grade temporal lobe gliomas. Methods: Twenty patients undergoing resection of temporal high-grade glioma between 2019 and 2022 underwent preoperative cognitive assessment using either the BLTT or a comprehensive neuropsychological battery routinely applied in temporal lobe epilepsy surgery. Results: Twelve patients (60%) underwent the BLTT, and eight (40%) underwent the standard neuropsychological test battery. The BLTT was feasible in all 12 individuals (0% drop-out), whereas only four of eight patients (50% drop-out) were able to complete the standard battery. The mean BLTT total score was 69.3 (SD 4.5), indicating impaired neurocognitive performance relative to normative data. The BLTT provided evaluable domain-specific measures across episodic memory, semantic memory, and executive functioning despite the high discontinuation rate observed with standard testing. Conclusions: The BLTT showed high feasibility for preoperative neurocognitive testing in patients with temporal high-grade gliomas. Its brief administration time and consistent applicability across relevant cognitive domains support its utility for routine neurocognitive assessment.

## 1. Introduction

High-grade gliomas, including glioblastoma, are highly malignant primary brain tumors with an incidence of approximately seven per 100,000 individuals [1]. Clinical presentation frequently includes seizures, language disturbances, memory problems, and attention and executive dysfunctions, particularly in temporal lobe tumors [2,3,4].

Neurocognitive functioning, especially executive functions, semantic memory, and episodic memory, is closely related to autonomy and quality of life, making its preservation a key objective in glioma surgery [5,6,7]. Existing studies report heterogeneous outcomes, which can be attributed to differences in test batteries and incomplete assessment of relevant domains [5]. Neurocognitive performance has further been shown to correlate with functional independence and prognosis in glioblastoma [8,9], establishing its relevance as a clinical parameter in glioma surgery.

The vast majority of brain tumor patients demonstrate measurable impairments in neuropsychological testing compared with matched controls [10]. In temporal gliomas, the affected hemisphere contributes to specific deficits: both sides may impair memory and learning, while semantic encoding is predominantly compromised in left temporal tumors [2,11]. Tumor grade further impacts neurocognitive function, with grade 4 gliomas showing the highest frequency of impairment [12].

Reliable assessment therefore requires feasible neuropsychological test batteries. Conventional batteries are time-consuming and often extensive in scope and require administration by trained neuropsychologists [13,14,15]. In neuro-oncological patients, the duration and complexity frequently limit feasibility, as many are unable to complete the full assessment. Their use is thus largely confined to research settings and rarely integrated into routine care. Therefore, clinically feasible neurocognitive assessment tools that capture domain-specific deficits are needed.

The Bonn Leistungs Tracking Test (BLTT), derived from the ViaCogScreen [16], is a short and standardized battery covering episodic memory, semantic memory, and executive functions. It was designed by the developer to remain applicable in patients with neurological deficits such as hemiparesis or visual impairment. This study evaluates the feasibility of the BLTT in patients with temporal high-grade gliomas by comparison with the application of an extensive neurocognitive test battery routinely used in temporal lobe epilepsy surgery at our epilepsy surgery center [17,18,19].

## 2. Materials and Methods

### 2.1. Patient Cohort

We conducted a retrospective analysis that included 20 patients with adult temporal high-grade glioma (glioblastoma or high-grade astrocytoma, reclassified based on the WHO 2021 classification) who were treated between 2019 and 2022 at the neuro-oncology center Bonn. Inclusion required neuroradiological suspicion of high-grade glioma, and grade 3 and 4 gliomas were subsequently included.

Twelve patients underwent assessment preoperatively with BLTT, and eight patients were tested with the in-house standard neurocognitive battery preoperatively [14,15]. Due to the retrospective study design, patients were assessed with either the BLTT or the standard neurocognitive test battery according to clinical routine. Parallel testing with both batteries was not performed.

The primary aim of this study was to evaluate the feasibility of the BLTT in patients with temporal high-grade glioma. Exploratory comparisons with the institutional standard neuropsychological battery were conducted to contextualize cognitive assessment outcomes.

### 2.2. Ethics

The study was approved by the local ethics committee of the University of Bonn (No. 2025-296-BO). Due to the retrospective study design, the requirement for informed consent was waived.

### 2.3. BLTT

The BLTT is the paper-and-pencil version of the ViaCogScreen [16], a German online computer-guided cognitive screening tool developed especially for older subjects. The tool has been developed by C. Helmstaedter for the company ViaMed GmbH in Stuttgart, which granted him the copyrights for the paper-and-pencil version. The tool integrates elements from the EpiTrack and the German Auditory Verbal Learning Test, detecting left temporal and mesiotemporal functions [20,21]. A complementary figural memory test is included, derived from the Diagnosticum für Cerebralschädigung (DCS-R) [22]. Consequently, the assessment covers the domains of executive functions, episodic memory, semantic memory, and figural memory, as all tasks are administered and responded to verbally, with no writing, reading, or drawing required. Testing is feasible even in patients with motor or visuospatial deficits. Normative data are available from 200 healthy controls aged 50–85 years, including data for repeated measures to support outcome monitoring. These norms are age-, gender- and education-adjusted. A total score (15–100 points; <76 = −1 SD, <72 = −2 SD, SD: Standard deviation) and three domain-specific subscores are generated: episodic memory (immediate, delayed, and recognition recall of 12 words), semantic memory (category fluency and semantic categorization tasks), and executive functions (backward digit span and phonemic fluency). For episodic memory, scores span between 2 and 14 points (<8 = −1 SD, <6 = −2 SD). The subscore for semantic memory is possible between 4 and 27 points (<17 = −1 SD, < 14 = −2 SD), while executive functions range from 7 to 41 points (<22 = −1 SD, <18 = −2D). When the figural memory task was administered, a fourth subscore was derived (recognition of target figures among distractors). The BLTT has been validated with suggested dementia and brain-damaged patients [16].

### 2.4. Standard Neurocognitive Test Battery

The standard neurocognitive test battery consists of multiple subtests and is frequently used in the assessment of patients with epilepsy based on temporal lobe pathologies [14,15]. The assessment includes verbal and figural memory, as well as attention, executive, visuo-spatial functions and language [23]. Subtests therefore assess right and left temporal functions and include the Verbaler Lern–und Merkfähigkeitstest (VLMT) for verbal memory [24,25,26], the DSC-R for figural memory [22], and the EpiTrack for attention and executive functions [20], and the Mehrfachwahl–Wortschatz Test (MWT-B) for intelligence [27].

### 2.5. Categorization of Subdomains

To enable comparison across batteries, results were converted into categorical scores ranging from 0 to 4 reflecting deviation from normative means: 0 = far below average (>2 SD), 1 = below average (>1 SD in >2 subtests), 2 = low average (>1 SD in one subtest), 3 = average (no deviation >1 SD), and 4 = above average (>1 SD in ≥2 subtests). This categorical approach was adopted from previously published neurocognitive studies [14,15]. These categories represent a standardized transformation of the underlying test results based on deviations from the respective normative means and standard deviations. Because the BLTT and the standard neurocognitive battery use different scoring systems and normative datasets, direct comparison of raw scores was not feasible. Therefore, this established categorical approach was used to facilitate cross-test comparison across both assessment batteries.

### 2.6. Additional Assessments

Depressive symptoms were assessed with the HADS (Hospital Anxiety and Depression Scale) in the BLTT group and with BDI-II (Becks–Depressionsinventar) in the standard battery group [28,29]. Due to the retrospective design, parallel application of both instruments was not available. The Karnofsky Performance Score (KPS) was used to classify patients according to their preoperative functional status [30].

### 2.7. Statistical Analysis

Baseline clinical characteristics were compared using the Mann–Whitney U test for continuous variables and the Chi-square test for categorical variables. Differences in completion rates were analyzed using Fisher’s exact test. Comparison of categorical scores between BLTT and the standard battery was performed using the Mann–Whitney U test. A two-sided *p*-value < 0.05 was considered significant. No correction for multiple testing was applied due to the exploratory character of the study. Analyses were performed with SPSS (IBM SPSS 29.0, IBM GmbH, Munich, Germany) and Prism (Version 10.5, GraphPad Software, Boston, MA, USA).

## 3. Results

### 3.1. Patient Characteristics

**Figure 1:**
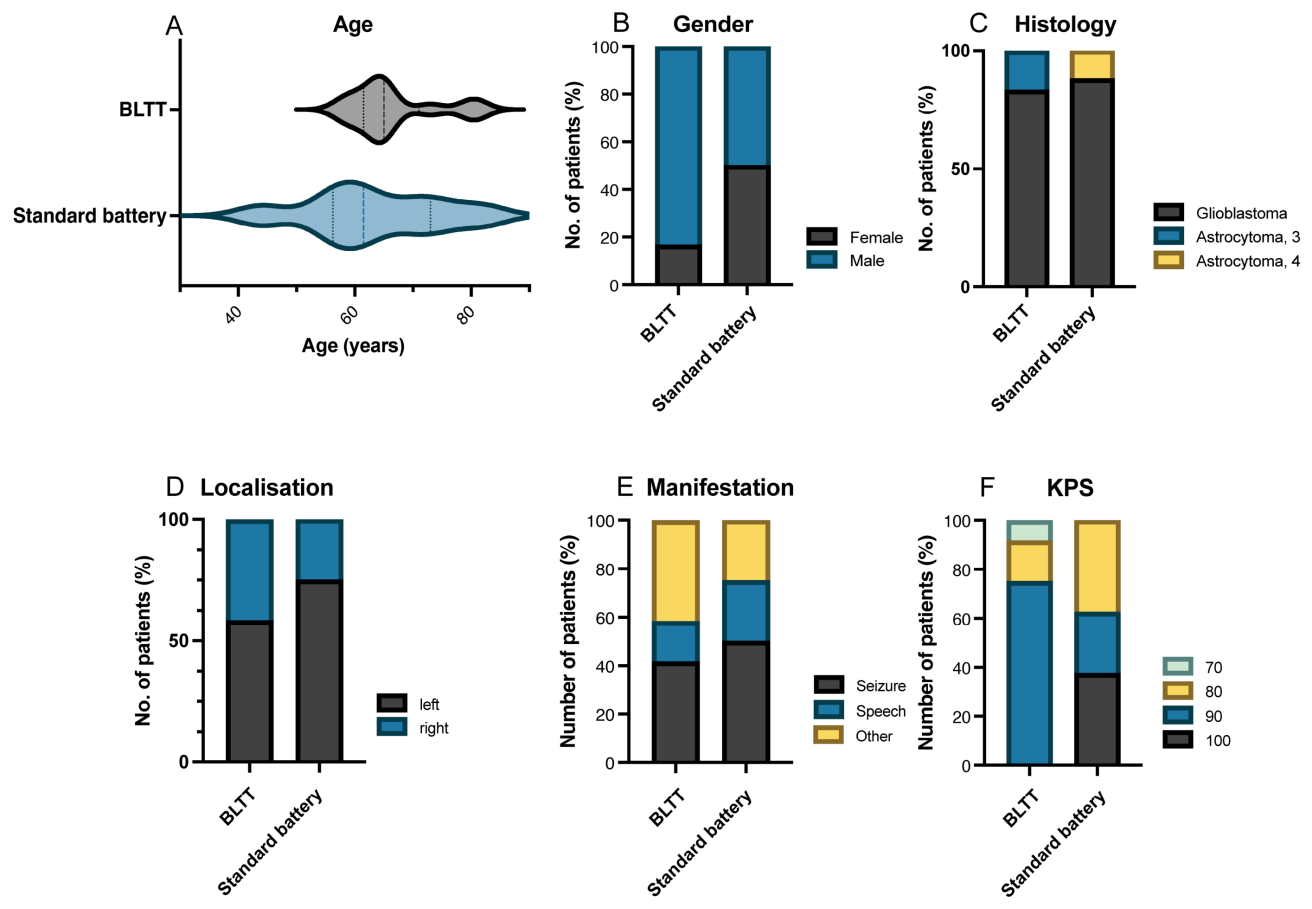
Baseline characteristics. (**A**) Age Distribution, (**B**) gender, (**C**) histology distribution, (**D**) localization, (**E**) first manifestation, (**F**) KPS preoperative. BLTT, Bonn Leistungs Tracking Test; KPS, Karnofsky Performance Index.

In total, 20 patients with temporal lobe gliomas underwent preoperative neurocognitive assessment, including 12 (60%) tested with the BLTT and 8 (40%) with the standard neuropsychological battery. The median age was 65 years in the BLTT group (IQR 62–71, IQR: interquartile range) and 61 years in the standard test group (IQR 56–73, *p* = 0.38) (Figure 1A). Sex distribution did not differ significantly between groups: nine male (75%) and three female (25%) patients in the BLTT group versus four male (50%) and four female (50%) in the standard group (*p* = 0.36, Figure 1B). The majority of patients were diagnosed with glioblastoma, IDH-wildtype CNS WHO grade 4 (BLTT: 10/12, 83%; standard: 7/8, 88%, *p* = 0.3), with the remaining patients diagnosed with IDH-mutant astrocytoma CNS WHO grade 3 (BLTT: 2/12, 17%) or astrocytoma, CNS WHO grade 4 (standard: 1/8, 12%) (Figure 1C). In the BLTT group, seven patients (58%) had a left-sided and five patients (42%) right-sided temporal glioma, while in the standard test battery group, six patients (75%) had left-sided and two patients (25%) right-sided temporal glioma (*p* = 0.64, Figure 1D).

The most common presenting symptom was seizures, reported in nearly half of patients in both groups (BLTT: 7/12, 58%; standard: 5/8, 63%, *p* = 0.85). Speech impairments, including amnestic aphasia or reduced fluency, occurred in 17% of BLTT patients (2/12) and 38% of standard group patients (3/8; *p* = 0.34). Additional symptoms included gait disturbance (BLTT: 3/12, 25%; standard: 1/8, 13%, *p* = 0.62), personality change (BLTT: 1/12, 8%; standard: 1/8; 13%, *p* = 0.76), and visuospatial impairments. Visual impairment due to hemianopsia was observed in 33% of BLTT patients (4/12) and 25% of standard group patients (2/8, *p* = 0.69) (Figure 1E).

The median preoperative KPS was 90% in both groups (BLTT: IQR 80–90; standard: IQR 80–100), indicating a high level of functional independence, with no significant difference between groups (*p* = 0.27) (Figure 1F). Screening for depressive symptoms revealed a mean HADS score of 9.8 (SD 3.8) in the BLTT group, suggestive of clinically relevant depression. In the standard test group, depressive symptoms were assessed using the BDI, with a mean score of 10.0 (SD 1.2), consistent with minimal depressive symptoms.

### 3.2. Feasibility

**Figure 2:**
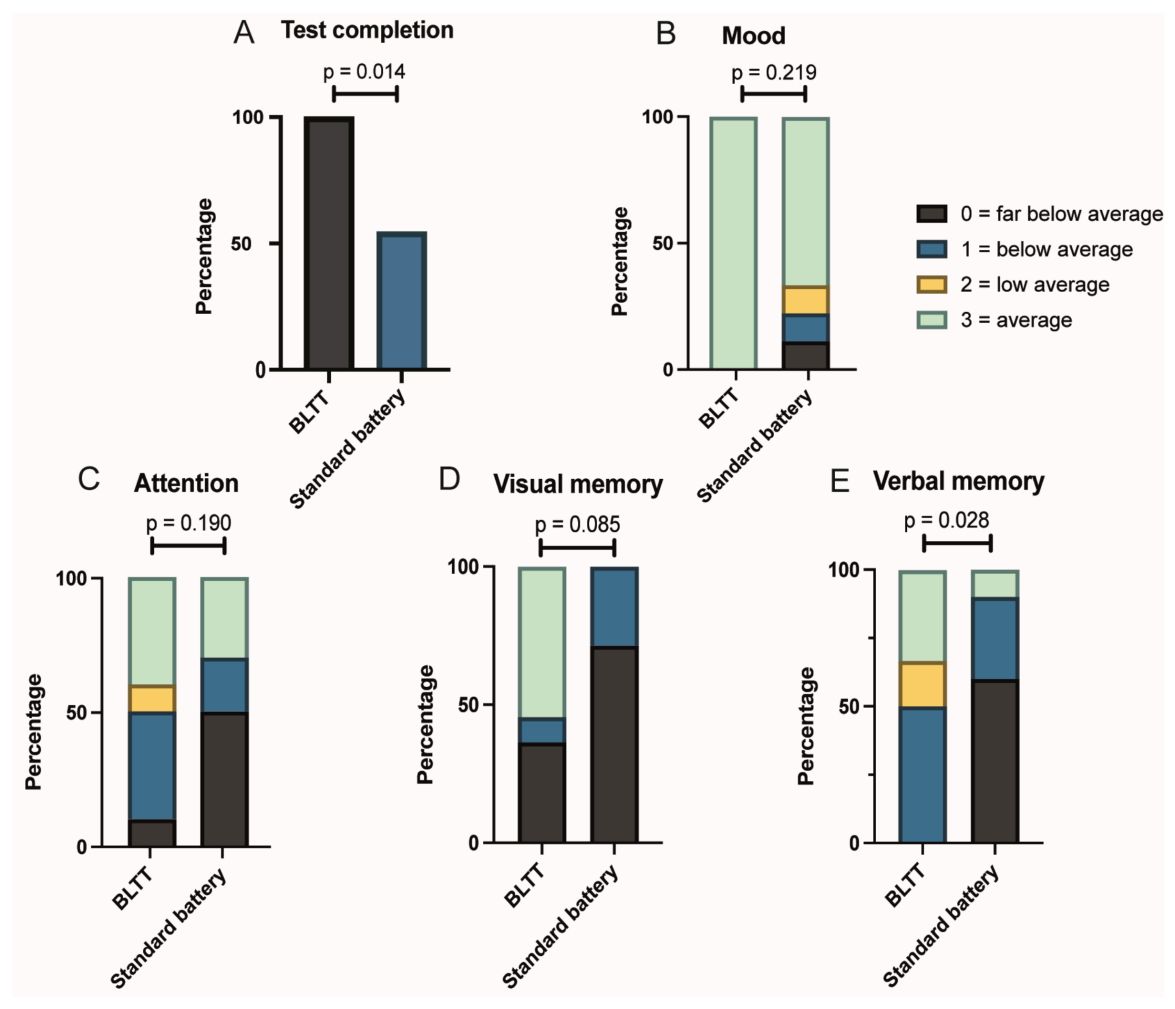
Comparison of the BLTT to the standard test battery. Stack plots with significance level for (**A**) test completion, (**B**) mood, (**C**) attention, (**D**) visual and (**E**) verbal memory. 0 “far below average”, 1 “below average”, 2 “low average, 3 “average”. BLTT, Bonn Leistungs Tracking Test.

All patients in the BLTT group completed the full assessment (100%), whereas 50% of patients in the standard test group discontinued prematurely (*p* = 0.014, Fisher’s Exact Test) (Figure 2A). The subtests most frequently discontinued were the MWT-B (global IQ, 2/8, 25%) and DSC-R (figural memory, 4/8, 50%). In one case, the standard test was aborted at the beginning, leaving no evaluable data. The BLTT, including figural testing, was completed with a median of 20 min (*n* = 9, IQR 17.5–20.0), compared to more than 60 min for the standard battery according to the institutional testing protocol, even without accounting for the frequent discontinuations.

### 3.3. Comparative Analysis

#### 3.3.1. BLTT Performance

The mean BLTT total score was 69.3 (SD 4.5), corresponding to approximately −2 SD relative to normative data (Table 1). Domain-specific analyses of the BLTT showed mean scores of 15.1 (SD 2.97) for semantic memory, 6.7 (SD 2.1) for episodic memory, and 19.5 (SD 3.5) for executive functions (Table 1). Compared with normative ranges (semantic memory: 4–27, episodic memory: 2–14, executive functions: 7–41 [16]), all domains were impaired by at least −1 SD, with episodic memory exceeding −2 SD and indicating the most pronounced deficit (Table 1). The strongest performance in the BLTT was observed in visuospatial functions (mean 2.4, SD 0.9), which were not assessed in the standard battery.

#### 3.3.2. Standard Test Battery Performance

In the standard test group, executive functions and attention, assessed with the EpiTrack, were unimpaired in 12.5% (1/8), impaired in 12.5% (1/8) of the patients and severely impaired in 75% (6/8) (mean 27.1, SD 2.2; maximum 37). The MWT-IQ averaged 111.2 (SD 8.7), within the normative range, although two assessments (25%) were terminated prematurely. The visual or figural memory task (DSC-R) was frequently aborted or yielded far-below-average scores (sum of the learned figures): only six of eight patients (75%) attempted the subtest, with two (25%) discontinuing early (mean: 8.7, SD 0.3, maximum 9), indicating below-average performance [24]. The VLMT revealed pronounced verbal memory impairment (based on standardized performance scores), with four patients (50%) scoring far below average, two (25%) below average, and only one (12.5%) achieving an average score.

### 3.4. Group Comparison

To allow comparison across batteries, results were standardized into domain subgroups (0–4: far below average to above average; Table 2). In the BLTT, mood was preserved (mean 3.0, SD 0), and a slightly lower score was observed in the standard battery (mean 2.3, SD 1.2, *p* = 0.29). On a group level, attention was impaired in the BLTT group (mean 1.8, SD 1.1) and in the standard battery (mean 1.1, SD 1.3; *p* = 0.27). After standardization of the domain scores, visual memory scores were descriptively lower in the standard battery (M = 0.25, SD = 0.5) than in the BLTT (M = 1.7, SD = 1.5), although this difference did not reach statistical significance (*p* = 0.14). Verbal memory scores were significantly higher in the BLTT (mean 1.8, SD 0.9) than in the standard battery (mean 0.7, SD 1.1; *p* = 0.028), although both groups demonstrated impairment relative to their respective normative data.

Individual analysis comparisons between groups were performed across mood, attention, visual and verbal memory (Table 2, Figure 2). Mood scores did not differ significantly between the two groups, although 25% of patients assessed with the standard battery scored in the low-to-far-below-average range (BLTT: mean 3.0, SD 0; standard battery: mean 2.3, SD 1.2; *p* = 0.29; Figure 2B). Attention was impaired in 63% of patients in the standard battery, who scored below or far below average, compared with 50% in the BLTT group (BLTT: mean 1.8, SD 1.14; standard: mean 1.1, SD 1.3; *p* = 0.27; Figure 2C). In the standard battery group, all patients scored below or far below average in the visual memory domain (mean 0.25, SD 0.5), whereas 44% of patients in the BLTT group fell into this range (mean 1.7, SD 1.49; *p* = 0.14; Figure 2D). Verbal memory scores were significantly higher in the BLTT group compared with the standard battery (BLTT: mean 1.8, SD 0.93; standard: mean 0.7, SD 1.1; *p* = 0.028). In the standard group, 85% of patients performed below or far below average, whereas in the BLTT group, 50% showed below-average results, with no cases far below average (Figure 2E, *p* = 0.028).

## 4. Discussion

Evaluation of neurocognitive functions is essential for prognosis in glioma patients, with preoperative status strongly predicting outcome and impairments linked to shorter survival [31,32]. It should therefore be part of routine clinical care. However, standard batteries are time-consuming and require expertise and are often not feasible in neuro-oncology. Here, we evaluated the clinical feasibility of a brief, multidomain cognitive screening approach designed to address some of the limitations of existing brief screening instruments like the MoCA in glioma patients [33]. Compared with comprehensive neuropsychological batteries, which may require between 1 and 8 h depending on the number and complexity of cognitive domains assessed [33,34], the BLTT can be completed within a median of approximately 20 min while still providing a multidomain cognitive assessment. Although brief screening instruments such as the Montreal Cognitive Assessment (MoCA) and the Mini-Mental State Examination (MMSE) require only approximately 10 and 5–10 min, respectively, their sensitivity for detecting subtle cognitive deficits in patients with glioma might be limited [33]. Thus, the BLTT may represent a feasible compromise between comprehensive neuropsychological assessment and brief cognitive screening in routine neuro-oncological practice. Patients with brain tumors assessed using the MoCA predominantly demonstrated impairments in naming and word fluency. Compared with the MMSE, the MoCA has shown greater sensitivity for detecting cognitive dysfunctions, while both tests were primarily developed for detection of global cognitive impairment [35]. Another brief cognitive screening instrument evaluated in patients with intracranial tumors is the Brief Cognitive Status Exam (BCSE), which demonstrated good feasibility but lower sensitivity than comprehensive neuropsychological assessment for detecting cognitive impairment across multiple cognitive domains [36]. The BLTT is derived from the ViaCogScreen, for which normative data, educational adjustment, and test–retest reliability have previously been established [16]. In addition, previous studies demonstrated good agreement with established neurocognitive assessments, including verbal memory measures such as the VLMT [16]. However, validation specifically in patients with glioma has not yet been established. Therefore, the present study should primarily be regarded as a feasibility study, whereas prospective studies are required to evaluate its concurrent validity and diagnostic performance in neuro-oncology. In this context, the BLTT may represent a feasible, time-efficient multidomain cognitive assessment that can be integrated into routine practice in neuro-oncology.

Importantly, the BLTT captures functions relevant for both left and right temporal as well as temporomesial regions by integrating verbal, figural memory, and executive function tasks [20,21,22]. This design addresses the known impact of tumor grade and location on neurocognitive functions, while tumor volume appears less predictive [12].

The choice of neurocognitive assessment reflected routine clinical practice rather than randomization. Patients initially underwent the comprehensive neuropsychological battery routinely used in our epilepsy surgery program. However, the high rate of premature discontinuation in this clinical setting highlighted the limited feasibility of prolonged testing in patients with high-grade gliomas and prompted the use of the shorter BLTT. Thus, the present study reflects a pragmatic clinical scenario in which feasibility was the primary outcome. Premature termination in the standard test battery was mainly related to prolonged testing time (>60 min) and patients being overwhelmed by standard test demands, compared with a median of 20 min for the BLTT. Importantly, both groups exhibited a comparable preoperative functional status, suggesting that differences in test completion were unlikely to be solely attributable to baseline clinical impairment. The most frequently discontinued subtest was the DSC-R, assessing figural memory and visuospatial functions relevant for detecting right temporal and temporomesial pathology [22]. Semantic memory tasks included in the BLTT may also be considered equivalent for crystallized intelligence, which is typically more stable than fluid cognitive functions such as executive performance. Interestingly, patients in the standard battery frequently discontinued the MWT-B, a vocabulary-based estimate of premorbid intelligence. This observation may reflect reduced tolerance for longer test procedures rather than a true impairment of crystallized cognitive abilities. Because the specific reasons for premature discontinuation were not prospectively documented, these findings should be interpreted with caution.

The present study was designed to evaluate the clinical feasibility of the BLTT rather than its diagnostic validity or equivalence to comprehensive neuropsychological assessment. Therefore, further prospective validation studies are required before conclusions regarding diagnostic performance can be drawn. Beyond feasibility, the consistent applicability of the BLTT has clinical implications. A short, standardized tool enables reliable preoperative assessment, even in patients with limited capacity to undergo extensive testing. It also facilitates longitudinal follow-up, allowing repeated assessments after surgery, radiotherapy, or chemotherapy. However, repeated neurocognitive assessment in patients with high-grade glioma remains challenging because disease progression, worsening neurological deficits, and loss to follow-up may limit repeated testing and introduce selection bias [37]. In contrast, neurological deficits such as hemianopia or hemiparesis do not necessarily preclude administration of the BLTT. Furthermore, repeated cognitive assessment requires instruments with minimal practice effects and established retest procedures. In this regard, test–retest norms for follow-up intervals of 6 months are available [16]. Moreover, the BLTT may serve as a standardized cognitive outcome measure in clinical trials, where cognitive endpoints are increasingly recognized as relevant for evaluating new therapies. Nevertheless, prospective longitudinal studies are required to further evaluate its performance throughout the course of glioma treatment.

Baseline characteristics were comparable between groups, including depressive symptom scores, which are known to influence survival in glioblastoma [8]. Despite similar clinical profiles, patients in the standard test battery group showed more frequent impairments and higher drop-out rates, which may indicate a floor effect of the standard battery and limit comparability and repeated assessments for outcome monitoring within this patient population. The more extensive standard neuropsychological battery demonstrated a higher frequency of deficits across domains. However, this likely reflects the greater sensitivity of longer and more detailed tests, which include additional components such as learning trials and visuoconstructive elements. At the same time, the prolonged testing duration resulted in a substantial drop-out rate, limiting the practical applicability of these assessments in patients with high-grade gliomas. In contrast, the BLTT consistently yielded evaluable results across relevant domains, even in patients without overt neurological deficits.

Although the present study focused on patients with temporal high-grade gliomas, the multidomain structure of the BLTT is not restricted to a single anatomical region. Nevertheless, prospective studies are required to evaluate its performance in patients with frontal, parietal, and occipital gliomas. Frontal lesions particularly affect executive function [38], and since the BLTT includes tasks assessing these domains, its use may therefore be applicable in this subgroup. Parietal and occipital lesions, which often cause visuospatial deficits [39], may also be suitable for assessment with the BLTT, as several components rely on recognition rather than complex visuoconstructive performance, allowing testing even in patients with partial visual field deficits such as hemianopia.

This study has several limitations. The retrospective design and small sample size restrict generalizability. The small sample size limits statistical power and increases the risk of Type II error, particularly for comparisons between the two study groups. No a priori sample size calculation or formal statistical power analysis was performed because of the retrospective exploratory nature of the study. Patients were not assessed with both batteries; therefore, no within-subject comparison was possible. Administering both batteries to the same patients would have substantially increased the testing burden and was therefore not considered clinically appropriate in this patient population. The study was designed to evaluate the clinical feasibility of the BLTT rather than its diagnostic validity. Consequently, no formal validation against a comprehensive neuropsychological reference standard was performed. Furthermore, no randomization was performed because the assessments were performed as part of routine clinical practice. Although baseline characteristics appear comparable between the groups, the limited sample size does not allow selection bias to be excluded. In addition, formal psychometric analyses, including inter-rater and intra-rater reliability assessments, were beyond the scope of this exploratory feasibility study and should be addressed in future prospective validation studies. Another limitation of the present study is the absence of standardized patient-reported outcome measures, such as the ECOG Performance Status or the EORTC QLQ-C30. Future studies should combine cognitive assessment with functional and quality-of-life measures to better characterize the clinical relevance of cognitive impairment in patients with glioma.

Furthermore, the effect of surgery on neurocognitive trajectories remains to be clarified. Current evidence suggests an initial postoperative decline with gradual recovery [40], whereas structured rehabilitation has shown benefits in temporal lobe epilepsy [41]. Additionally, future research should address internal stability and test–retest reliability to establish its psychometric robustness.

## 5. Conclusions

The present study suggests that the BLTT is a feasible tool for assessing neurocognitive function in patients with temporal high-grade gliomas. It achieved a higher completion rate than the in-house comprehensive battery used as the standard assessment in temporal lobe epilepsy surgery. The BLTT enabled multidomain cognitive assessment across attention, semantic memory, and episodic memory within a short administration time. Its short duration and high feasibility support its use as a practical approach for routine neurocognitive assessment in neuro-oncological patients. Future studies should validate the BLTT in larger cohorts, apply it preoperatively and longitudinally, and combine it with patient-reported outcomes to establish its role in clinical practice.

## Figures and Tables

**Table 1:** Score values of patients tested with the BLTT. Mean test result of total test results and subtest results.

	BLTT			
	n	Mean	SD	Range
Total Score	12	69.3	4.5	61–74
Core semantic memory	12	15.1	2.97	11–19
Core episodic memory	12	6.7	2.06	3–10
Core executive functions	12	19.5	3.5	13–23

BLTT, Bonn Leistungs Tracking Test; n, number; SD, Standard deviation.

**Table 2:** Group analysis of results of BLTT and Standard test battery in terms of categorical results.

	BLTT			Standard Battery			
	n	Mean	SD	n	Mean	SD	*p*-Value
Mood	12	3	0	6	2.3	1.2	0.29
Attention	10	1.8	1.1	7	1.1	1.3	0.27
Visual Memory	11	1.7	1.5	4	0.25	0.5	0.14
Verbal Memory	12	1.8	0.9	7	0.6	1.1	0.028 *
Visuo-spatial	12	2.4	0.90	0	/	/	/

Score categories 0 up to 4 in every subgroup. Statistically analyzed with Mann–Whitney U-Test. 0 “far below average”, 1 “below average”, 2 “low average, 3 “average”, 4 “above-average”. * *p*-value < 0.05. BLTT, Bonn Leistungs Tracking Test.

## Data Availability

Restrictions apply to the availability of these data due to privacy restrictions.
